# Dr. Bernardino Calabrese

**Published:** 1906-09

**Authors:** 


### OBITUARY.

Dr. Bernardino Calabrese, of Buffalo, died at his office, No. 47 Scott street, August 16, 1906, after a brief illness. He was born in Samfele, Province of Potenzo, Italy, in 1838. He was graduated from the University of Naples in 1861, and was one time mayor of his native town.

Dr. Calabrese came to Buffalo about 15 years ago and enjoyed a large private practice, besides being official physician to the Italian consulate. He was an honorary member of several Italian societies in this city and also of the Scientific Society in Palermo, Sicily. He is survived by one son, Thomas Calabrese, of Buffalo.

